# Supplementary material for: Stabilizing gold nanoparticles for use in X-ray computed tomography imaging of soil systems
Source: R Soc Open Sci. 2019 Oct 16;6(10):190769. doi: 10.1098/rsos.190769 (PMC6837195; doi:10.1098/rsos.190769)
Supplement: Supplementary Information [file rsos190769supp1.docx]

# Supplementary Information

## 1 Buffer Power Approximation for Silica and Alumina

**Methodology:**

Governing Equation

In order to determine a lower bound estimate for the buffer power of AuNPs in silica and alumina, we constructed a mathematical model to simulate the experimental results shown in **Figure S2**. To represent the experimental set up of the flow column system as shown in **Figure 1**, we used a one-dimensional advection-diffusion equation. This takes the form

|  | ($\phi+b)\frac{\partial c}{\partial t}=D_{e}\frac{\partial^{2}c}{\partial x^{2}}-V\frac{\partial c}{\partial x}, \boldsymbol{x\inΩ,}$ | (1) |
| --- | --- | --- |

where $\phi$[m^3^ m^-3^] is the porosity of the porous media, *b* is the buffer power of the PEG-AuNPs, $V$ [m^3^ s^-1^ m^-2^] is the volumetric flux of water, $D_{e}$ [m^2^ s^-1^] is the effective diffusion coefficient for the AuNPs adjusted for impedance caused by the porespace steric conditions in the silica and alumina, and $\Omega$ is the 1D domain that represents the column shown in **Figure 1**.

Since the flow column system was fully saturated, we assumed the effective diffusion coefficient takes the power form and used the expression by Nye (32, 33) *i.e.*,

| $D_{e}=D\phi^{3},$ | (2) |
| --- | --- |

where $D$ is the diffusion coefficient of the PEG-AuNPs in free liquid. The diffusion coefficient $D$ was derived from the Stokes-Einstein equation for the diffusion of spherical particles through a liquid that has a low Reynold’s number. The Stokes-Einstein equation is given by

| $D=\frac{Tk}{6\pi\eta r},$ | (3) |
| --- | --- |

where $T$ is the absolute temperature, $k$ is the Boltzmann constant, $\eta$ is the suspension viscosity and $r$ is the hydrodynamic radius of the particle.

Boundary Conditions

For the boundary condition at the top of the flow column system, we noted that an O-ring and cotton wool were placed at the top of the column to prevent flush-back of the PEG-AuNPs during the addition of 0.5 ml of UHQ water above the O-ring (**Figure 1**), *i.e.*, the AuNPs were insulated within the column and not able to leave. Hence, to replicate this condition mathematically, we imposed a no flux condition so that the AuNPs were contained within the domain, *i.e.*,

| $D_{e}\frac{\partial c}{\partial x}-Vc=0, \boldsymbol{x\in}\partial\boldsymbol{Ω}_{\boldsymbol{T}}\boldsymbol{,}$ | (4) |
| --- | --- |

where $\partial\boldsymbol{Ω}_{\boldsymbol{T}}$ is the boundary at the top of the domain.

Since we aimed to capture the results seen in **Figure S2**, *i.e.*, there is no movement of the AuNPs over 24 hours, we noted that the AuNPs did not reach the base of the domain and bound to the particle surface at the top of the system. Since the domain is sufficiently large to avoid any AuNPs reaching the base of the domain, we imposed another no flux boundary condition, *i.e.*,

| $D_{e}\frac{\partial c}{\partial x}-Vc=0, \boldsymbol{x\in}\partial\boldsymbol{Ω}_{\boldsymbol{B}}\boldsymbol{,}$ | (5) |
| --- | --- |

where $\partial\boldsymbol{Ω}_{\boldsymbol{B}}$ is the boundary at the base of the domain. To validate the insulating condition, we checked there was zero concentration on $\partial\boldsymbol{Ω}_{\boldsymbol{B}}$ throughout the simulation when the model results matched the experimental results.

Initial Conditions

The domain length was 0.04 m and an initial condition of PEG-AuNPs was imposed in the first 0.002 m of the domain to replicate the experimental system, *i.e.*,

| $c\vert_{t=0}= \left\{ \begin{matrix} 1 \\ 0 \end{matrix} \right.\begin{matrix} 0 m\boldsymbol{\leq x\leq}0.002 m \\ 0.002 m\boldsymbol{<x\leq}0.04 m \end{matrix} .$ | (6) |
| --- | --- |

**Results:**

A lower bound estimate of the buffer power was determined by increasing $b$ in the model until a similar rate of immobilisation was observed in the simulation results as captured in the experimental results (**Figure S2**). It was demonstrated that when a buffer power of 70 or above is applied the solution curves at 0 hours and 24 hours have a difference of 75 µm (**Figure S4**). Therefore, these solution curves indicate that the PEG-AuNP pulse had become immobilised at the surface of the silica and alumina. Consequently, the approximate lower bound for the buffer power of silica and alumina on PEG-AuNPs was determined to be $b\approx70$.

# List of Supplementary Tables

[**Supplementary Table S1.** Parameter values used for the convection diffusion model to simulate the movement of AuNPs through the silica and alumina column. 3](#_Toc4492471)

# Supplementary Tables

**Supplementary Table S1.** Parameter values used for the convection diffusion model to simulate the movement of AuNPs through the silica and alumina column.

| **Parameter** | **Symbol** | **Value** | **Reference** |
| --- | --- | --- | --- |
| Porosity of soil | $\phi$ | 0.4 | (40) |
| Diffusion coefficient of AuNPs | $D$ | 7x10^-12^m^2^ s^-1^ | - |
| Effective diffusion coefficient of AuNPs adjusted for soil porosity | $D_{e}$ | 4.5x10^-13^m^2^ s^-1^ | - |
| Volume flux of water | $V$ | 5.0x10^-8^m s^-1^ | (30) |
| Buffer power of silica/ alumina | $b$ | 70 | - |

# List of Supplementary Figures

[**Supplementary Figure S1.** Horizontal image slices from XCT images of silica and/ or alumina displaying the accumulation of AuNPs on the silica and alumina surfaces. These images are from: a) a column of silica and alumina mixed b) a column of alumina c) a column of silica. Gold nanoparticle accumulation on the surface of the silica and alumina particles are visible bright bands on the exterior of the particles. Regions of prominent accumulation are marked with orange rings and specific examples are highlighted by the orange arrows. Scale bars are 1 mm in length. 4](#_Toc528763172)

[**Supplementary Figure S2.** XCT images of silica and/ or alumina confirming the accumulation of AuNPs on the very surface of the silica and alumina in the columns. Right: histograms display the maximum grey levels down the column for a) mixed silica and alumina b) alumina c) silica. Immediately after AuNP addition is displayed in blue and 24 hours after AuNP addition is displayed in orange. The gold nanoparticle induced peak is indicated in green. Left: a representative vertical slice through the image stack and the orange arrow displays the region from which data was extracted. 5](#_Toc528763173)

[**Supplementary Figure S3.** Gold nanoparticles accumulated on the surface of silica or alumina particles captured using scanning electron microscopy (SEM) imaging. Gold nanoparticles are visible as the brighter regions. The four images are: the surfaces of alumina particles at 4,000× magnification (a), and silica particle surfaces at 400× (b), 2,000× (c) and 30,000× (d) magnification, respectively. 6](#_Toc528763174)

[**Supplementary Figure S4.** The results of the 1D advection-diffusion model for the movement of AuNPs down a flow column with an AuNP pulse placed atop 4cm of silica and alumina. The buffer power applied in this simulation was b=70. The difference between the solution curves at 0 hours (blue dots) and 24 hours (orange dashes) was 75 µm indicating that the buffer power of 70 was sufficient to simulate the immobilisation of AuNPs on the silica and alumina surface. 7](#_Toc528763175)

# Supplementary Figures


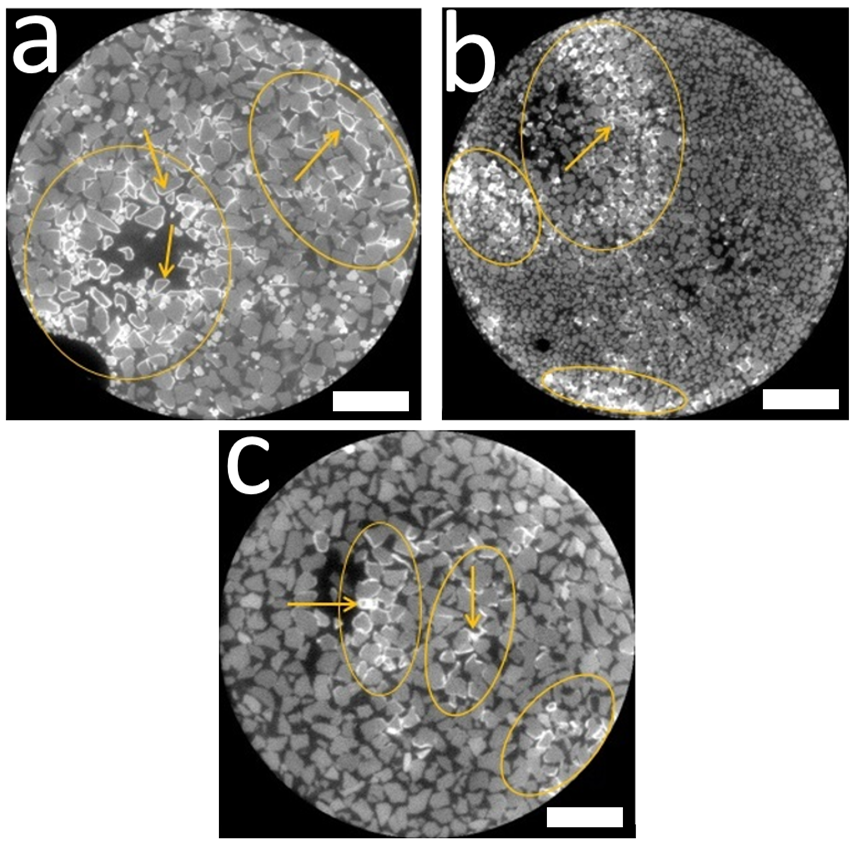


**Supplementary Figure S1.** Horizontal image slices from XCT images of silica and/ or alumina displaying the accumulation of PEG-AuNPs on the silica and alumina surfaces. These images are from: a) a column of silica and alumina mixed b) a column of alumina c) a column of silica. Gold nanoparticle accumulation on the surface of the silica and alumina particles are visible bright bands on the exterior of the particles. Regions of prominent accumulation are marked with orange rings and specific examples are highlighted by the orange arrows. Scale bars are 1 mm in length.


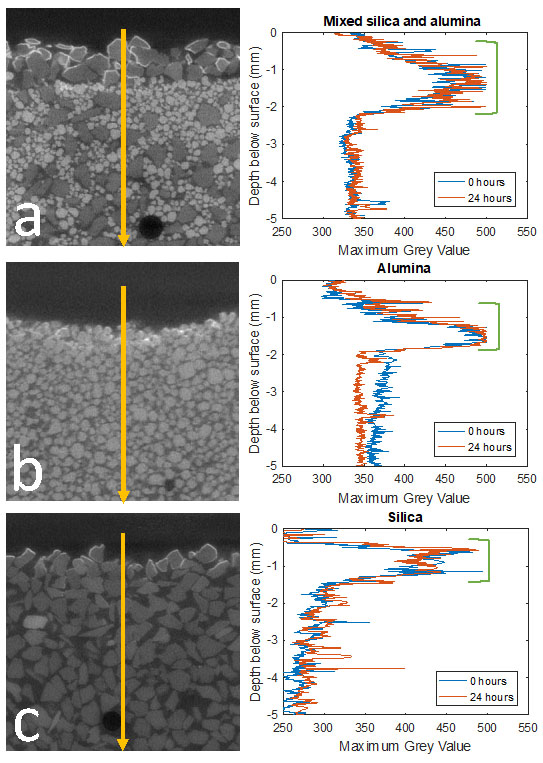


**Supplementary Figure S2.** XCT images of silica and/ or alumina confirming the accumulation of PEG-AuNPs on the very surface of the silica and alumina in the columns. Right: histograms display the maximum grey levels down the column for a) mixed silica and alumina b) alumina c) silica. Immediately after AuNP addition is displayed in blue and 24 hours after PEG-AuNP addition is displayed in orange. The gold nanoparticle induced peak is indicated in green. Left: a representative vertical slice through the image stack and the orange arrow displays the region from which data was extracted.


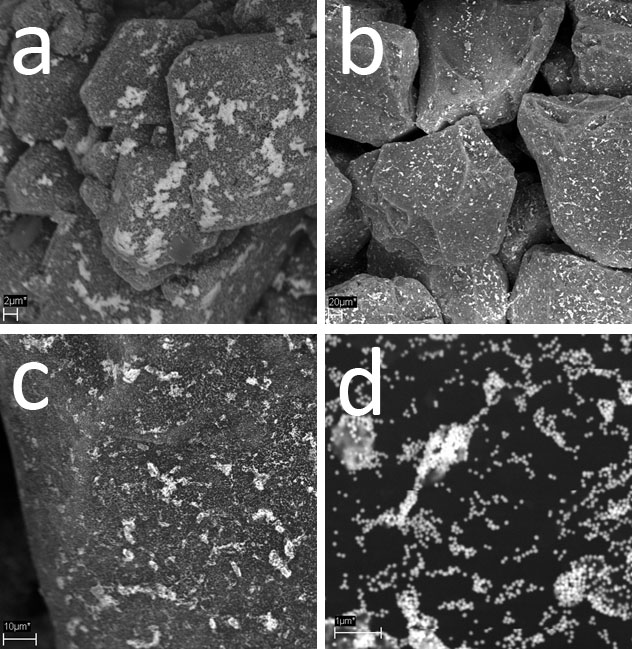


**Supplementary Figure S3.** Gold nanoparticles accumulated on the surface of silica or alumina particles captured using scanning electron microscopy (SEM) imaging. Gold nanoparticles are visible as the brighter regions. The four images are: the surfaces of alumina particles at 4,000× magnification (a), and silica particle surfaces at 400× (b), 2,000× (c) and 30,000× (d) magnification, respectively.

**Supplementary Figure S4.** The results of the 1D advection-diffusion model for the movement of AuNPs down a flow column with an AuNP pulse placed atop 4cm of silica and alumina. The buffer power applied in this simulation was b=70. The difference between the solution curves at 0 hours (blue dots) and 24 hours (orange dashes) was 75 µm indicating that the buffer power of 70 was sufficient to simulate the immobilisation of AuNPs on the silica and alumina surface.
