# Supplementary figures and images for: Stabilizing gold nanoparticles for use in X-ray computed tomography imaging of soil systems
Source: R Soc Open Sci. 2019 Oct 16;6(10):190769. doi: 10.1098/rsos.190769 (PMC6837195; doi:10.1098/rsos.190769)

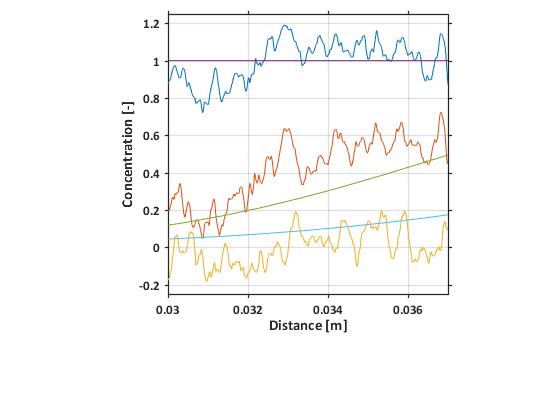

Supplement: AU concentration fit [file rsos190769supp20.jpg]
